# Design of cohort studies in chronic diseases using routinely collected databases when a prescription is used as surrogate outcome

**DOI:** 10.1186/1471-2288-11-36

**Published:** 2011-04-01

**Authors:** Sara Lodi, James Carpenter, Peter Egger, Stephen Evans

**Affiliations:** 1 London School of Hygiene and Tropical Medicine, London, UK; 2 GlaxoSmithKline - Stockley Park, Uxbridge, UK

## Abstract

**Background:**

There has been little research on design of studies based on routinely collected data when the clinical endpoint of interest is not recorded, but can be inferred from a prescription. This often happens when exploring the effect of a drug on chronic diseases. Using the LifeLink claims database in studying the possible anti-inflammatory effects of statins in rheumatoid arthritis (RA), oral steroids (OS) were treated as surrogate of inflammatory flare-ups. We compared two cohort study designs, the first using time to event outcomes and the second using quantitative amount of the surrogate.

**Methods:**

RA patients were extracted from the LifeLink database. In the first study, patients were split into two sub-cohorts based on whether they were using OS within a specified time window of the RA index date (first record of RA). Using Cox models we evaluated the association between time-varying exposure to statins and (i) initiation of OS therapy in the non-users of OS at RA index date and (ii) cessation of OS therapy in the users of OS at RA index date. In the second study, we matched new statin users to non users on age and sex. Zero inflated negative binomial models were used to contrast the number of days' prescriptions of OS in the year following date of statin initiation for the two exposure groups.

**Results:**

In the unmatched study, the statin exposure hazard ratio (HR) of initiating OS in the 31451 non-users of OS at RA index date was 0.96(95% CI 0.9,1.1) and the statin exposure HR of cessation of OS therapy in the 6026 users of OS therapy at RA index date was 0.95 (0.87,1.05). In the matched cohort of 6288 RA patients the statin exposure rate ratio for duration on OS therapy was 0.88(0.76,1.02). There was digit preference for outcomes in multiples of 7 and 30 days.

**Conclusions:**

The 'time to event' study design was preferable because it better exploits information on all available patients and provides a degree of robustness toward confounding. We found no convincing evidence that statins reduce inflammation in RA patients.

## Background

Routinely collected data - such as databases of health care insurance claims or the General Practice Research database (GPRD) - have become a very important source of information for studying secondary effects of drugs [1-3]. They are useful as they usually provide information on the health care history of many patients for relatively long periods of time. Also, since the data have already been collected, they allow investigation of the secondary effects of drugs relatively quickly and cheaply in comparison to randomised trials or prospective studies.

However, such routinely collected data have been relatively rarely used in studies examining secondary effects of drugs on progression/exacerbation of chronic diseases [e.g. [4-8]]. As such databases are not compiled with epidemiological research in mind, they usually provide no information on clinical endpoints which do not result in a new diagnosis or hospitalisation. Hence, information on chronic conditions where the outcome of interest is not a new recorded diagnosis or hospitalisation is often poor. Nevertheless, in certain situations, the prescription of a drug used to treat the symptoms of a chronic disease may be regarded as a surrogate or "substitute" for the outcome of interest [8,9]. For example, anti-inflammatory drugs for flare-ups in autoimmune disease or anti-depressant drugs for depression.

Using prescriptions as a surrogate or "substitute" for unmeasured endpoints raises a number of design and analysis issues, which to our knowledge have not been fully explored. First, we need a clinically and contextually appropriate definition of how prescription of the surrogate marker drug represents the unmeasured endpoint. Then, having chosen a surrogate prescription, we have a modelling choice: time to surrogate initiation/cessation or quantitative surrogate use.

If we choose the former, and take prescription as a binary outcome, this can be analysed either by logistic regression or by Cox regression using the time to the event as outcome. It could also be analysed as a recurrent event for multiple prescriptions, though this assumes that a second or subsequent prescription has the same meaning as the first, which may be doubtful. Furthermore, the event may be one of two types: for those who are on the surrogate drug at the start of follow-up, the event of interest would be stopping the surrogate drug, while for those not on it at the start, the event of interest is the first prescription of it.

If we take the latter, we need to summarise the amount of the surrogate drug used in a specific period. This could be cumulative dose or number and duration of prescriptions where dosage information is limited or varies very little. In both approaches the drug used as surrogate outcome might be contraindicated in some patients, and the information in the record may be insufficient to determine whether it should be contra-indicated. This complicates statistical modelling, which should reflect the mix of contra and non-contra indicated patients in the database.

Taking as a motivating example investigating the possible ameliorative effect of statin use on Rheumatoid Arthritis flare-up, the aim of this paper is to describe and critically compare the analysis options. This leads to general recommendations concerning approaches to studying secondary drug effects using routinely collected data in chronic diseases. We have previously reported the main results from our data [8], but here we focus on the choice of the design and analysis of this type of study.

In Section 2 we give more detail on the research question, and describe the routinely collected data we analysed to investigate it. Section 2.1 describes a time-to-initiation and time-to-cessation of surrogate drug analysis and Section 2.2 an analysis based on the quantity of the surrogate drug use. The results are presented in Section 3. In Section 4 we critically evaluate the various approaches, and also consider further under which conditions a drug prescription can be regarded as an appropriate surrogate or "substitute" for an endpoint for the prognosis of a chronic disease. Finally, we outline the implications for similar future studies.

## Methods

For our motivating example we investigate the suggestion that statins may have anti-inflammatory effects [10-14] and, in particular, that they may prevent or delay an inflammatory flare-up in patients with Rheumatoid Arthritis (RA). As RA is a long-term chronic disease with a complex and only partially understood aetiology, characterised by recurrent flare-ups, a large routinely collected database is a natural source of information.

### Data source

We used data from the LifeLink insurance claim database which is available for research use [15]. This database, whose former name was PharMetrics Patient Centric Database, is collected, standardised and maintained by IMS Health [16]). This US medical and pharmacy claims database recorded insurance claims on 1.8 million employees, their dependents and retirees within an employer health care insurance scheme. The database reports basic information on eligibility for care, demographic characteristics, and both in- and out-patient medical and pharmacy claims. The study was approved by the Ethical Committee of the London School of Hygiene and Tropical Medicine. Within the database we identified a study population of 37477 patients with at least one medical claim for RA. The first RA claim is regarded as a proxy for RA diagnosis.

A new diagnosis is not recorded unless there is a claim, and while the recording of a claim within the database is not necessarily the first date of a diagnosis it is the best that can be done. We have noted [8] that claims that occur early in the longitudinal record of a patient are likely to be prevalent cases (and made allowance for likely prevalent cases).

### Choice of a surrogate marker of inflammatory status

In order to explore the anti-inflammatory effects of statins on RA patients a measure of inflammatory status is needed. As direct information on inflammatory status is not available in Lifelink, we treated the prescription of oral steroids (OS) (prednisone and its derivates) as a surrogate marker or 'substitute' for inflammatory status. Initiation of OS therapy is a plausible marker for the start of an inflammatory bout since such OS therapy was commonly prescribed to keep inflammation under control during the period when these data were collected. Furthermore, because of the side effects of long-term use of OS, such treatment is usually discontinued as soon as the inflammation is under control. Having made this choice, as discussed in the introduction there are two approaches to the analysis: model time to initiation/cessation of OS therapy or model the quantity of use of OS therapy. We now describe these in more detail.

### Cohort study for time to change in inflammation

**Figure 1:**
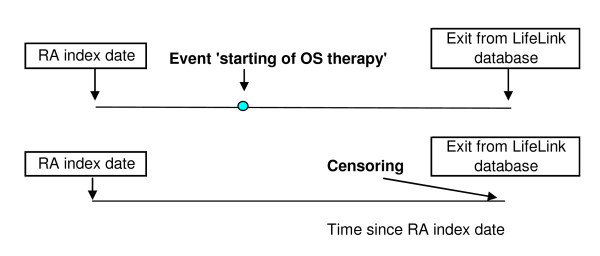
**Definition of outcome event and censoring in the sub-cohort I (non-users of OS at RA index date)**.

**Figure 2:**
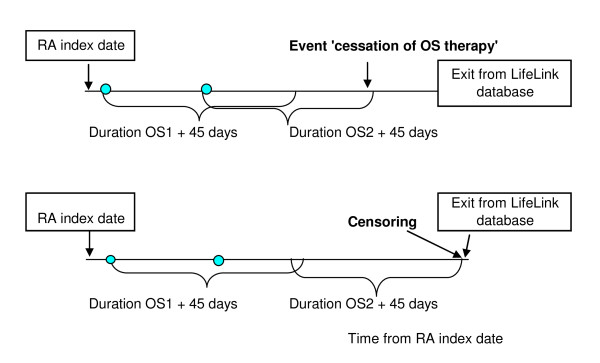
**Definition of outcome event and censoring in the sub-cohort II (users of OS at RA index date)**.

We used an unmatched cohort study where initiation and cessation of OS therapy were regarded as markers for an increase and decrease in the underlying/unmeasured RA inflammatory process. We described this study design in detail previously [8]. Briefly, the eligible patients were split into two mutually exclusive sub-cohorts based on whether they were not, or were, using OS in a time window of RA index date +/-30 days (Figures 1, 2). The rationale for this time window was that recorded dates of prescriptions and diagnosis may not be exact. Sub-cohort I included the patients not on OS therapy at RA index date. The outcome event was time to initiation of OS therapy defined as the first prescription of OS (Figure 1). Sub-cohort II included the patients who were on OS therapy at RA index date. The outcome event for this was time to cessation of OS therapy, defined as the date of the end of the first OS prescription which was not followed by a subsequent prescription within 45 days (Figure 2). To account for the progressive nature of the disease, the time origin was defined as RA index date in both sub-cohorts. As patients who start statin therapy might stop it and restart it later, we defined statin exposure as a time-varying covariate. The research hypothesis was that if statins have anti-inflammatory effects, then patients who use statins should both have a reduced risk of initiating OS therapy and an increased risk of cessation of OS therapy compared to patients who do not take statins.

Separate Cox proportional hazards models were used to evaluate the association between time-varying exposure to statins and i) initiation of OS therapy in the non users of OS at RA index date (sub-cohort I) and ii) cessation of OS therapy in the users of OS at RA index date (sub-cohort II). The models were adjusted for gender, an indicator for RA incident cases (as time between enrolment and first RA diagnosis > 6 months), medical coverage (full versus partial) and health plan (indemnity versus preferred provider organisation [PPO] scheme) --since it is possible that type of insurance could affect likelihood of prescriptions both for statins and OS) -- and, to account for the secular trend in use of statins and changes in RA management over the period of the study, calendar year of RA index date (1992 to 2002). Models were adjusted by co-medications (non steroidal anti-inflammatory drugs [NSAID], disease modifying anti-rheumatic drugs [DMARD], ACE inhibitors, thiazide, loop diuretics, warfarin, beta blockers, angiotensin II blockers, oral anti-diabetics and insulin) and co-morbidities (diabetes mellitus, autoimmune disease other than RA, osteoarthritis, asthma, chronic obstructive pulmonary disease [COPD], peptic ulcer, cardiovascular disease) and oral steroids prescribed before RA index date. More parsimonious models were selected by retaining covariates with p-value < 0.1 in the full model adjusted for all the covariates. The models were stratified by age at RA index date (20-50, 51-70, and 70-88) as this was found necessary for the assumption of proportional hazards [17].

### Matched cohort with amount of oral steroid prescribed as outcome

We used a matched cohort study where new users of statins (exposed group) were matched on the date of the initiation of statin therapy 1 to 1 to patients without any record of statin use in the previous year (unexposed group) by sex and exact age. The statin index date was defined as the calendar date of first statin prescription for an exposed patient and the same date for the matched unexposed patient.

Patients were included if they had at least one medical claim for RA before statin index date, were aged more than 20 years at first RA diagnosis (to exclude patients with juvenile arthritis), and had at least 365 days of membership of the database before the statin index date (to ensure that a patient is truly a new user of statins and to leave enough time to detect pre-exposure co-morbidities and co-medications).

**Figure 3:**
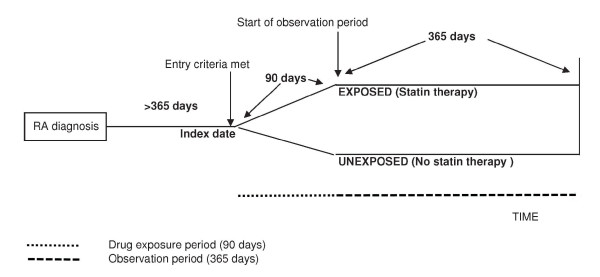
**Structure of study cohort matched by sex and age**.

As Figure 3 shows, we defined the observation period as the 365 days following the 'run-in' period of 90 days--after which a patient can be considered fully exposed to a statin [18].

Then, we analysed the data from this matched cohort taking two different definitions of the outcome.

First, the outcome was defined as a binary variable indicating whether the patient had used OS therapy during the observation period (yes = 1, no = 0). The research hypothesis was that if statins have an anti-inflammatory effect, then patients who started statins are less likely to use OS therapy during the observation period than the patients who did not have a statin. To account for the matched nature the study design and increase precision of the estimates we used conditional logistic regression [19,20].

Second, the outcome was defined as the proportion of days under OS therapy (as indicated in the pharmacy records) in the observation period. The research hypothesis was that if statins have an anti-inflammatory effect, statin users will have on average fewer days of OS therapy than non-users. Patients in the unexposed group were censored if they initiated statins during the observation period. As a quick tool to compare the proportion of days under OS therapy in the observation period in the two exposure groups, we used a paired Wilcoxon test. As this test does not allow for adjustment of covariates, we then used regression modelling for count data. As we expected that a number of patients would have received no OS therapy during their follow up, possibly due to contra-indication, the zero inflated negative binomial model was used to account for the outcome variable having an excess of zeros [21]. This model assumes that the data come from a mixture of two processes. In our setting, the first is whether or not a patient receives OS therapy. The second, if they received OS, how many days they are prescribed (0,1,2), etc.

The model was adjusted for prescription of medications before the index date (disease modifying anti-rheumatic drugs [DMARDs], non steroidal anti-inflammatory drugs [NSAIDs], digoxin, potassium sparing diuretic, loop diuretic, thiazides, heparin, beta blockers, angiotensin II inhibitors, ACE inhibitors, clopidogrel bisulfate, warfarin, anti-diabetics, insulin and oral steroids) and morbidities before the index date (cardiovascular disease, diabetes mellitus, chronic obstructive pulmonary disease [COPD], asthma, peptic ulcer disease and osteoarthritis). In order to select a more parsimonious model, for the binary part of the model we selected the covariates which resulted in a p-value less than 0.1 from a multivariable logistic regression. For the negative binomial component, we used the covariates which resulted in a p-value less than 0.1 from a multivariable negative binomial model. We included an offset [i.e., log_e_(length of patient follow-up)] to allow for the length of follow-up time spent on OS therapy.

Finally, a quick way of assessing whether matching has achieved balance and overlap is to plot histograms of the propensity scores, ie the probability a patient receives a statin given everything was observed before the treatment, across exposed and the unexposed groups [22]. For each patient, the propensity score was estimated by fitting a multivariable logistic regression of statin exposure (yes = 1, no = 0) on all baseline covariates together with the first terms interactions and age to the square. Overlap in the distribution of the propensity score does not ensure that all predictors included in the model are similarly matched, but they provide some indication that their distributions have close balance across the two groups

## Results

Table 1 reports the estimated association between statin use and OS prescription from each of the analyses described above after adjustment for potential confounders. More detailed results are described below and in Tables 2 and 3 and Tables 1 and 2 in Lodi et al [8].

**Table 1:** Summary of methods and results after adjustment for potential confounders

Study design	Outcome	Statistical methods	Estimate of effect of statin exposure *	P
Matched cohort	Use of OS in the observation period	Conditional logistic regression	Odds ratio 1.064 (0.879,1.288)	0.523
	Number of days on OS therapy in the observation period	Zero inflated negative binomial model	a) Odds ratio 1.025 (0.900,1.169)	0.704
			b) Rate ratio 0.879 (0.758,1.021)	0.091

Unmatched cohort ^	Time to initiation of OS therapy	Cox model	Hazard ratio 0.956 (0.901,1.015)	0.14
	Time to cessation of OS therapy	Cox model	Hazard ratio 0.954 (0.866,1.051)	0.34

*Baseline reference: not exposed to statins

^Results presented in reference [8]

**Table 2:** Matched cohort study.

	Odds ratio	95% confidence interval		P
Statin exposure	1.064	0.879	1.288	0.523
Fibrate	1.167	0.905	1.504	0.233
Ace inhibitors	0.961	0.911	1.014	0.144
Digoxin	1.005	0.919	1.098	0.92
Warfarin	1.078	0.951	1.223	0.242
Loop diuretic	1.01	0.888	1.149	0.879
NSAID	1.014	0.976	1.054	0.472
OS	2.158	1.881	2.477	<0.001
Thiazide	0.98	0.909	1.058	0.612
DMARD	1.083	1.042	1.126	<0.001
Potassium sparing	1.029	0.905	1.169	0.665
Beta blockers	1.029	0.958	1.105	0.433
Angiotensine	1.071	0.948	1.211	0.268
Clopidogrel	1.091	0.888	1.341	0.408
Hyperlipidemia	0.805	0.607	1.067	0.131
Transient Ischaemic attack	1.153	0.706	1.882	0.569
Osteoarthritis	1.2	0.924	1.558	0.172
Angina	0.918	0.707	1.192	0.522
Asthma COPD	1.638	1.263	2.124	<0.001
Myocardial infarction	1.299	0.853	1.98	0.223
Diabetes	0.455	0.271	0.764	0.003
Hypertersion	1.147	0.485	2.714	0.754
Stroke	0.744	0.502	1.101	0.139
Peptic Ulcer	0.81	0.408	1.608	0.546
Cerebral Heamorragea	1.098	0.509	2.367	0.811
Heart failure	0.569	0.172	1.889	0.357

Results from the conditional logistic regression adjusted for morbities and medications before the index date

**Table 3:** Matched cohort study.

	*Rate ratio*	*95% Confidence interval*		*P*
Statin	0.879	0.758	1.021	0.091
Asthma COPD	0.815	0.676	0.983	0.032
Osteoarthritis	0.854	0.715	1.019	0.081
Hyperlipidemia	0.808	0.665	0.981	0.031
Hypertension	0.855	0.72	1.017	0.076
Peptic ulcer disease	0.763	0.538	1.082	0.13
OS	1.356	1.148	1.602	<0.001
DMARDs	1.545	1.282	1.862	<0.001
NSAID	0.757	0.637	0.899	0.002

	***Odds Ratio***	***95% confidence interval***		***P***

Statin	1.025	0.900	1.169	0.704
Diabetes mellitus	1.369	1.107	1.690	0.004
Asthma COPD	0.829	0.703	0.977	0.026
Hyperlipidemia	1.428	1.210	1.685	<0.001
Hypertension	1.171	1.010	1.359	0.037
OS	0.386	0.332	0.450	<0.001
DMARD	0.433	0.363	0.517	<0.001
Loop diuretics	1.242	0.938	1.644	0.13
Anti-diabetics	1.895	1.334	2.691	<0.001

Results from the zero inflated negative binomial model. Parsimonious model including all covariates (morbidities and medications at the index date)

with p-value less than 0.1. The model has two components. Bottom: logistic model component predicting the probability of NOT having OS. Top: negative binomial model component predicting the rate of OS duration in an observation period of length 365 days for those patients who have OS.

### Cohort study based on time to change in inflammation

After splitting patients according to their use of OS at RA index date we found 31451 non users of OS at RA index date and 6026 users of OS at RA index date. Amongst the 31451 non users of OS at RA index date, during a follow-up of 99749 person years, 11402 (36%) began OS therapy during their follow up, and the median time to initiation of OS was 6.8 years. Amongst the users of OS at RA index date 5490 (91%) ceased OS therapy during their follow up of 4410.3 person-years. The median time to OS cessation was 0.37 years.

In the sub-cohort of non users of OS at RA index date, a hazard ratio <1 indicates a protective effect of statin use on initiating OS therapy consistent with a possible anti-inflammatory effect. As shown in Table 1, the adjusted hazard ratio for statins of initiating OS therapy was 0.956 (95% CI 0.901,1.015), hence statin use only slightly and statistically non-significantly reduced the likelihood of start of OS therapy. Baseline records for diseases treated with OS like autoimmune disease, asthma and COPD were associated with an increased likelihood of initiating OS therapy [8]. On the other hand, cardiovascular diseases such as cerebral haemorrhage and stroke, diabetes and hypertension were associated with a decreased likelihood of initiating OS therapy.

In the sub-cohort of users of OS at RA index date the outcome was cessation of OS therapy. Hence, a protective effect of a covariate is indicated by a hazard ratio >1 consistent with an anti-inflammatory effect. The adjusted exposure hazard ratio of cessation of OS therapy for statin use was 0.954 (95% CI 0.866, 1.051), indicating that statin use had only a small and statistically non-significant 'prolonging' effect on OS use. Cardiovascular diseases such as angina, hypertension, and to a lesser extent, myocardial infarction were significant predictors of cessation of OS therapy [8].

### Matched cohort study with amount of OS prescription outcome

**Figure 4:**
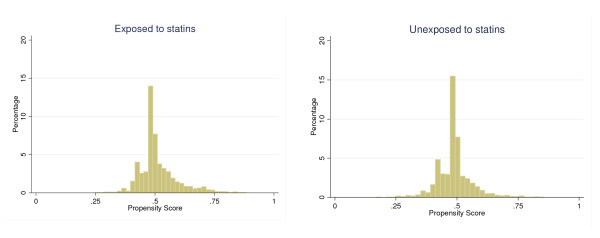
**Matched cohort study**. Distribution of the propensity score by exposure group.

**Figure 5:**
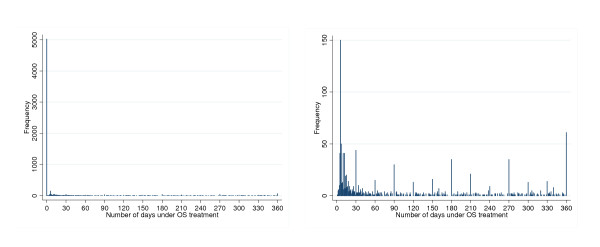
**Matched cohort study**. Distribution of number of days on OS therapy in the observation period: left panel- all patients; right panel: excluding patients who did not initiate OS therapy.

The matching algorithm found 3144 patients newly exposed to statins after RA diagnosis and 3144 unexposed patients matched in terms of age and sex. The contribution to follow up was 3142 person-years and 3057 person-years in the exposed and unexposed group, respectively. 372 patients entered the cohort in the unexposed group but became statin users in the observation period. The distribution of the propensity score, ie the probability of starting a statin given the observed covariates, is shown in Figure 4. There was a slight tendency for the exposed patients to have higher propensity score values. Nevertheless, the distributions show an overall overlap. 80% of patients (5034 out of 6288) had no OS prescription in the observation period (Figure 5a). Figure 5b shows that among patients with non zero outcome, there is, not surprisingly, digit preference for 7 and multiples of 10 and.

When we used a binary outcome OS prescription was slightly but not significantly less common among those who were exposed to a statin (600/3144 patients) compared with those who were not exposed (654/3144 patients) - matched-pair crude odds ratio f 0.901 (95% CI 0.795,1.019, p = 0.093). When we used a multivariable conditional logistic regression to account for potential confounders (first row, Table 1), we found an adjusted matched-pair odds ratio 1.064 (0.879,1.288, p = 0.523).

We then analysed the data using the quantitative outcome duration of OS therapy in the observation period. Due to the large proportion of patients with 0 duration outcome, median [interquartile range] duration was 0 0 0 in both exposed and unexposed group. The results from the unadjusted negative binomial model (rate ratio 0.792, 95% CI 0.608,1.030, p = 0.082) and the Wilcoxon matched-pairs signed-ranks test for of the difference in distribution of the paired differences in duration on OS therapy in the observation period (p = 0.036) both indicated some evidence of differences. Results from the adjusted zero inflated negative binomial model are presented in Table 1. The statin exposure odds ratio of 1.025 (95% CI 0.900,1.168) estimated from the logistic component predicting the probability of having OS (a) suggests that exposure to statins does not affect whether or not a patient is likely to be prescribed with OS therapy in the observation period. The statin exposure rate ratio of 0.88 (95% CI 0.79,1.06) estimated from the negative binomial component predicting the rate of OS duration in the observation period for those patients who have OS estimates (b) shows that among patients who received OS, the data are consistent with the hypothesis that statin exposure does not affect the rate of OS duration, though there is a hint that they could be protective. The full model is presented in Table 3. Here, history of diabetes, hyperlipidemia, hypertension and oral anti-diabetics have an increased odds of not being prescribed OS in the observation period, while patients with history of OS, asthma or COPD, DMARD are more likely to have OS therapy in the observation period.

## Discussion

We have described and illustrated two approaches to cohort study design that can be used to explore the possible secondary effects of a drug on a chronic disease using routinely collected databases. This work was motivated by the study of the anti-inflammatory effects of statins in RA patients using the LifeLink database: we explored possible anti-inflammatory effect of statins in Rheumatoid Arthritis where oral steroid (OS) prescription was chosen as a surrogate or "substitute" outcome for flare up. None of the proposed study designs provided convincing evidence of a protective effect for statins. A full discussion on the exposure definition and limitations of using routinely collected LifeLink database for studying the anti-inflammatory effects of statins is reported elsewhere [8]. Here our focus is on the pros and cons of the two approaches, which are well illustrated by our study.

In the first approach, the design was based on time to change OS therapy status, as a surrogate for the true outcome of interest, change in inflammation. The working hypothesis was that if statins have anti-inflammatory effects, then patients who use statins should both have a reduced risk of initiating OS therapy and an increased risk of cessation of OS therapy compared to similar patients who do not take statins.

In the second approach, we matched new statin users (when they initiated therapy) to patients who were not on statin therapy 1 to 1 on age and sex. The working hypothesis was that if statins have anti-inflammatory effects, then statin users should require OS for a shorter period compared to non users.

We now argue that the time-to-initiation/cessation approach is superior to the matched cohort study being both statistically more straightforward and epidemiologically more convincing. Consider the following points.

First, an awkward complicating issue when using the second approach is that a non-trivial proportion of patients will not be eligible for the surrogate medication (in our case OS therapy) for a variety of reasons that are often difficult to model. For example in our study 80% of patients did not have OS therapy in the observation period; this is an unidentified mix of those who are, and are not, eligible. By contrast, the first approach of splitting the cohort into two sub-cohorts (those under, and not under, OS therapy at RA index date) neatly sidesteps this first issue. A further advantage in the time-to-initiation/cessation approach both groups are modelled separately, allowing different-specific-adjustment for confounding.

Second, the Cox proportional hazard model (with appropriate use of stratification) provides a flexible framework for modelling the time-to-event (initiation or cessation of OS therapy in our example). In our case, this fitted the data satisfactorily. By contrast, with the second approach we must model the typically awkward data on surrogate (here OS) therapy use. Indeed, not only are such data often awkward to model, they are markedly affected by measurement error. In our study, both the extent of impossible doses removed in the data cleaning process, and the marked digit preference at 10 and 30 days, illustrate this.

Third, separately modelling the time to initiation/cessation of surrogate therapy means that - if the results are consistent - we can be more confident they are not substantially confounded. This is because the approach splits the patients into two sub-cohorts at index date, in which different mechanisms are at work, and these are independently followed-up and analysed. The analysis can then readily account for the fact that some predictors for initiation of surrogate therapy may differ from those for cessation of surrogate therapy. Further unmeasured confounders will likely work differently in the two cohorts. Thus, if -- as in our case -- the two sub-cohorts give compatible conclusions (i.e. one hazard ratio>1 and one hazard ratio<1), then we can be more confident the results are not substantially confounded.

Fourth, by splitting the available patients into non users/users of the surrogate therapy at index date the first approach also provides some degree of robustness with regard to contraindication of the surrogate therapy. For example in our study, patients who have a very low chance of being prescribed with OS because of contraindications are more likely to be part of the subcohort I, than in subcohort II. If a similar effect is found in both sub-cohorts, we are more confident the conclusions are robust.

Fifth, in our matched study only a minority of the available patients (20%) had at least one OS prescription in the observation period. Hence, the statistical power was reduced. This is typical in our experience. While a natural next step would be to increase the matching ratio, by attempting to match 1:M, unfortunately the gain in power/efficiency from each additional match quickly declines [23,24]. Further increasing the number of matches inevitably involves widening the matching 'calipers' so that the benefit of matching is reduced.

Sixth, and finally, in the time to event approach, patients can naturally contribute repeated episodes to the analysis (eg time to first initiation, then time from initiation to cessation etc.). We investigated this approach for these data, but it did not alter the substantive conclusions.

In summary, methodological and epidemiological considerations strongly favour the first approach.

Lastly, the use of surrogate end points has been criticised and it has been suggested that surrogate outcomes should not been used without validation studies proving that the treatment effect of interest is the same in the surrogate and in the endpoint of clinical importance [25,26]. While validation studies are clearly desirable, we argue such a dogmatic approach is not always appropriate or helpful. In the case of our motivating example, while a validation study showing that the effect of statins in RA patients is the same on the inflammatory status and on the risk of initiation/cessation of OS therapy has not been conducted, we think that OS prescription can be regarded as an appropriate surrogate marker or "substitute" for inflammatory status, because many if not most patients will start OS therapy quickly in response to an RA flare-up and most will stop OS therapy after a flare-up ends - not least because the side effects of OS therapy. We believe that in similar settings -- such as prescription of antidepressants as a surrogate marker for depression, prescription of anti-histamine for allergies, anti-inflammatory drugs for autoimmune disease flare-ups -- the use of prescriptions as surrogate markers (or substitutes) for the otherwise unrecorded status of chronic diseases is key to realising the full potential of routinely collected databases.

## Conclusions

We have compared, contrasted and illustrated two different approaches to the use of routinely collected databases for assessing the secondary effect of exposure on the prognosis of chronic disease. We used a surrogate therapy for the status of the chronic disease. The first approach was based on time to initiation/cessation of the surrogate therapy. The second approach used a matched cohort study to model the use of surrogate therapy. We have elucidated and illustrated six reasons why the first approach is preferred from both a statistical and epidemiological perspective. We therefore advocate the time-to-event approach as the method of choice for such studies.

## Competing interests

PE is an employee of and has shares in GlaxoSmithKline.

JC has provided consultancy and training to GlaxoSmith-Kline, Pfizer and Boehringer Ingelheim, Novartis and Bayer Pharmaceutical companies, and has received PhD studentship funding from GlaxoSmithKline and Amgen.

SL's PhD project was funded by GSK.

No other conflict of interest to declare

## Authors' contributions

SL, SE, JC conceived the study and participated in its design. SL performed the statistical analyses and prepared a draft of the manuscript. All authors contributed to interpreting the data and to a critical revision of the manuscript. PE contributed with the acquisition of the data

## Pre-publication history

The pre-publication history for this paper can be accessed here:

http://www.biomedcentral.com/1471-2288/11/36/prepub
